# The effect of complete feed to carcass characteristics and meat quality of male Bali cattle fattened in West Timor, Indonesia

**DOI:** 10.14202/vetworld.2020.2515-2527

**Published:** 2020-11-26

**Authors:** Paulus Klau Tahuk, Oktovianus R. Nahak, Gerson F. Bira

**Affiliations:** Department of Animal Science, Faculty of Agriculture, Universitas Timor, Kefamenanu, Timor Tengah Utara, East Nusa Tenggara, Indonesia

**Keywords:** Bali cattle fattened, carcass characteristics, complete feed, *Gliricidia sepium*, meat quality

## Abstract

**Aim:**

This research aimed to know the effect of the use of complete feed on Bali cattle fattening performance seen from the carcass characteristics.

**Materials and Methods:**

The cattle employed in this research were 12 male Bali cattle aged between 2 and 2.5 years old based on the teeth estimation. The average initial body weight of the cattle during the research was 181.50±16.51 kg. The complete feed contained *Gliricidia sepium*, natural grass, ground corn, bran pollard, and rice bran which have been compiled into three types of ration of T1, T2, and T3. The T_1_ ration contained standard crude protein (CP) and high energy (11% CP; 72% total digestible nutrient [TDN]), and T_2_ contained medium protein and high energy (13% CP; 72% TDN), while T_3_ ration contained high protein and high energy (%15 CP; 72% TDN).

**Results:**

The meat percentage of T2 and T3 was relatively the same, but higher than T1 (p<0.05). The bone percentage and meat: A bone ratio of T2 was higher than T1; in contrast, and T3 was relatively the same with T2 and T1 (p<0.05). The weight of slaughter, carcass percentage, and weight of meat, bone, and fat were relatively the same among the treatments.

**Conclusion:**

The application of complete feed with protein source from *G*. *sepium* with CP and TDN of 13 and 72%, respectively, can improve carcass percentage and meat: A bone ratio of male Bali cattle fattening. The treatments have not had a positive effect on slaughter weight (kg), hot and cool carcass weight (kg), meat and fat weight (kg), fat percentage, and non-carcass (kg).

## Introduction

Beef cattle (Bali cattle) fattening in Timor Island (West Timor) has been done by farmer/breeder in the hereditary area. The farmers/breeders have depended on their life need fulfillment by raising cattle in addition to agricultural activities. Therefore, Bali cattle rising have become an unseparated part of the local community life [1]. Even though it has been an unseparated part of the local community life, cattle fattening done by the farmer/breeder still encounters issues regarding the lack of feed availability in terms of quality and quantity [2]. Report given by Tahuk *et al*. [3] showed that the feed quality has lower feed quality, of which the amount is not sufficient for the feed need. It causes the increase of daily body weight gain (average daily gain [ADG]) of Bali cattle fattening in dry season becomes lower, which only reaches 0.30 kg/day, while ADG of the cattle in the wet season is higher by achieving 0.51 kg/day [3]. This indicates that season significantly affects the quality of feed, which eventually also affects the cattle growth. When viewed from the percentage of carcasses produced, the Bali cattle that are kept on the smallholder farm can reach 54.07-55.61%; but the percentage of meat produced only ranged between 65.19% and 68.78% [4].

The fluctuation issue of feed between the wet season and dry season needs to be solved so that the productivity of Bali cattle fattening by farmer/breeder in East Nusa Tenggara can be improved, whether it is in the dry season or wet season. Furthermore, the breeder can perform the cattle fattening all year without encountering worries of the existence of cattle performance decrease. One of the solutions offered to overcome the lack of feed is complete feed production by utilizing forage feed, which is sufficiently abundant in the wet season. If the complete feed production can be developed well, then there will be sufficient feed ingredient stock with sufficient nutrition to meet the cattle needs during the lack of food. According to Sivajanani and Sinniah [5], technology complete feed has the potential to provide highly nutritious feed to livestock under emergency situations created by natural calamities. Production of these types of feeds is very much important for enhancing the productivity of animals and for making use of the available low-cost feed material. The use of complete feed ingredient is beneficial to be applied since it can be presented to the cattle at once both the forage and concentrate so that it can increase the feed palatability as well as minimalizing the feed selection characteristics of the cattle, especially for the forage feed ingredients which needs adaptation from the cattle including *Gliricidia sepium*. According to Tahuk *et al*. [6], if the use of *G*. *sepium* as fresh basal feed and protein source reaches 46%, it can decrease the feed palatability and improve the cattle selection nature. It causes the nutrition obtained is not maximal and negatively affects the cattle growth performance. Therefore, the utilization of *G*. *sepium* in complete feed production is expected to decrease the selection nature, increase the palatability so that the cattle performance can be improved. The role of the complete ration is to provide a blend of the feed ingredients, including roughages without giving any choice to the animal for the selection of specific ingredients [7]. This is due to the complete feed provision, which can input balance food nutrients needed by cattle so that it can trigger the cattle growth. According to Tahuk and Bira [8], a complete feed is one of the solutions that can be taken to meet the needs of livestock. Complete feed has complete nutritional content to meet livestock needs. To produce complete feed, feed manipulation with correct ration formulation is one of the essential factors that must be done in livestock raising. However, this has not been done much by farmer/breeder in Indonesia, especially Timor Island (West Timor) since most of them still raise the cattle traditionally [1,9].

As such, the introduction of applicative feed technology, such as complete feed to farmer/breeder is important to be done to trigger the raising of livestock activities. Furthermore, manipulating protein levels to be different from the feed energy content are important to be done aiming to know the correct protein and energy level for the carcass characteristics and meat quality of Bali cattle fattening. This is due to the balanced nutrient especially the protein and energy, which can assure the cattle productivity.

Therefore, this research aimed to know Bali cattle fattening performance through complete feed seen from the carcass characteristics including the slaughter (final) weight; the weight and percentage of the carcass; weight and percentage of meat, bone and fat; weight and percentage of non-carcass; and meat-bone ratio. The other objectives are to know the physical and chemical quality of male Bali cattle which obtain complete feed.

## Materials and Methods

### Ethical approval

This type of study does not require ethical approval. However, cattle were handled without any harm.

### Time, location, cattle, and feed

The research was conducted in the experimental cage of the Faculty of Agriculture of Universitas Timor from April to September 2019, consisting of stages of preparation, data collection, analysis, and report. Feed sample was analyzed in the Chemistry Laboratory of Feed, Faculty of Animal Husbandry, Universitas Nusa Cendana Kupang. Cattle slaughtering for carcass data collection and its component were done in the slaughterhouse of CV. Aldia Kupang. The measurement of the physical and chemical quality of meat, as well as meat cholesterol, was carried out at the Faculty of Animal Science, Universitas Gadjah Mada, Yogyakarta; each in the Meat Science and Technology Laboratory and the Feed Biochemistry Laboratory.

The cattle employed were as many as 12 male Bali cattle aged between 2 and 2.5 years based on the teeth estimation. The general average initial body weight of the cattle during the research was 181.50±16.51 kg, with average initial body weight detail of treatment of T1 was 175.25±9.18; T2 was 184.75±13.94, and T3 was 184.50±25.36.

The complete feed contained *G. sepium*, natural grass, ground corn, bran pollard, and rice bran, which have been compiled into three types of ration of T1, T2, and T3 (Table-1). The T1 ration contained standard crude protein (CP) and high energy (CP by 11% and total digestible nutrient [TDN] by 72%) and T_2_ contained medium protein and high energy (CP by 13% and TDN by 72%), while T_3_ ration contained high protein and high energy (CP by 15% and TDN by 72%) (Tables-2 and 3). Besides, mineral premix was also added to meet the cattle needs.

**Table-1:** Nutrient content of feed ingredients composition and ration of complete feed for Bali cattle fattening.

Feed ingredients	Nutrient content									

	DM	OM	CP	EE	CF	CHO	NFE	GE		ME
			
	(%)	(% DM)						MJ/kg.DM	Kkal/kg.DM	Kkal/kg.DM
Field grass	88.986	77.388	5.318	0.805	28.221	71.266	43.045	13.892	3307.69	2123.13
*Gliricidia sepium*	85.260	76.397	21.377	3.403	11.137	51.617	40.479	15.272	3636.16	2848.33
Ground corn	85.950	83.012	9.609	8.967	3.059	64.437	61.378	16.535	3937.01	3750.37
Bran pollard	87.165	82.663	16.648	3.329	6.902	62.685	55.784	16.015	3813.19	3302.04
Rice bran	89.691	77.062	10.444	8.181	15.103	58.436	43.334	15432	3674.28	2960.72
Ration										
T1	88.047	80.466	14.866	5.542	14.070	60.058	45.988	15.884	3781.88	3014.04
T2	87.355	79.633	15.589	5.058	14.167	58.986	44.820	15.709	3740.13	2951.32
T3	87.464	70.582	16.671	3.397	14.863	59.514	44.651	15.495	3689.39	2843.36

DM=Dry matter, OM=Organic matter, CP=Crude protein, EE=Extract eter, CF=Crude fiber, CHO=Carbohydrate, NFE=Nitrogen free extract. Calculated through the equation of NFE=[100−(Ash content+CF level+EE content+CP content)] %, GE=Gross energy, ME=Energy metabolism, T1=Complete feed ration containing 11% CP, 72% TDN, T2=Complete feed ration containing 13% CP, 72% TDN, T3=Complete feed ration containing 15% CP, 72% TDN

**Table-2:** Comparison of feed ingredients composition and ration of complete feed for Bali cattle fattening.

Feed type	T1 ration	T2 ration	T3 ration
Field grass (%)	27	27	27
*Gliricidia sepium (%)*	10	20	31
Ground corn (%)	34	28	20
Bran pollard (%)	15	15	15
Rice bran (%)	14	10	7
Total	100	100	100

**Table 3:** Formulation of complete ration of research for Bali cattle fattened.

Feed ingredients	Nutrients content of the ration	

	Crude protein (%)	Total digestible nutrients (%)
T1 ration		
*Gliricidia sepium*	2.60	7.80
Ground corn	3.70	30.60
Rice bran	1.00	7.10
Bran pollard	2.40	11.10
Total	11.60	72.20
T2 ration		
Field grass	1.90	15.70
*Gliricidia sepium*	5.20	15.60
Ground corn	3.10	25.20
Rice bran	0.70	5.10
Bran pollard	2.40	11.10
Total	13.30	72.60
T3 ration		
Field grass	1.90	15.70
*Gliricidia sepium*	8.10	24.20
Ground corn	2.20	18.00
Rice bran	0.50	3.50
Bran pollard	2.40	11.10
Total	15.00	72.50

Before the application of complete feed, the ration feed ingredients in the form of *G*. *sepium* and natural grass was dried for approximately 2-3 days. Furthermore, it was ground using a grinding machine with a sieve size of 10 mm. The corn kernels as digestible carbohydrate sources were also digestible using the grinding machine on a sieve size of 5 mm. The corn that has been ground was then mixed with other feed ingredients of rice bran and bran pollard evenly until it became homogeny. The complete feed that has been prepared was then provided to the cattle.

### Research design

This research was carried out using a complete randomized design (CRD) of the unidirectional pattern. As many as 12 male Bali cattle were divided into three groups consisting of two groups obtained ration treatment while the other one acted as the control. The number of cattle in each group was four cattle for T1, T2, and T3 ration. Cattle were placed in an individual cage with a size of 150×200 cm, equipped with a place for eating and drinking. The cattle adaption toward the complete feed took 21 days (3 weeks), while the data collection stage spent 11 weeks. The provision of ration during the data collection was performed twice a day which was in the morning at 08.00 central Indonesian times and afternoon at 16.00 central Indonesian times. The drinking water was provided *ad libitum*.

### Variable measurement

The variable measured in this research included; (1) slaughter weight, (2) the weight and percentage of carcass, (3) weight and percentage of meat, bone, and fat, (4) weight and percentage of non-carcass, (5) meat-bone ratio; and (6) meat quality including the chemical and physical meat quality, and meat cholesterol.

The slaughter weight is the cattle weighing result before being slaughtered and has been fasted for ±24 h. During the fasting period, the drinking water was provided *ad libitum*. Hot or fresh carcass weight was obtained from the carcass weighing result before being withered in the chilling room. The percentage of hot carcass obtained from the comparison of hot and carcass weight and slaughter weight times 100%. The cool carcass weight (kg) was obtained after a hot carcass was withered in the chilling room for ±12 h.

Carcass component weight is the weight from each carcass main component after being separated, which includes beef, trim fat, and bone. The percentage of carcass components is the calculation result based on the weight comparison of each carcass component (meat, trim fat, and bone) with the cool carcass weight times 100%.

### Data collection and research procedure

Data collection was done for 6 months according to the length of the research conducted. The feed provided was weighed and recorded along with the remaining feed to know the amount of feed consumption. Feed nutrient composition was known by taking 500 g of feed sample, which was then dried until it reached the constant weight and 220 g concentrate, which was ground using Willey mill with a sieve hole of 1 mm to analyze the nutrient content in the laboratory.

#### The determination of the carcass characteristics

The cattle weighting to obtain bodyweight were done on each cattle in the primary research. Further weighting was done each week (7 days) to adjust the amount of feed to be given in addition to know the daily body weight gain and cattle growth pattern. To know the carcass production and its component, the cattle were slaughtered at the final raising phase. Before the slaughtering, the cattle were fasted for ±12 h to decrease the content of the digestive tract. Slaughtering was done after the cattle slaughter weight was measured.

#### The determination of the meat chemical quality

The chemical quality of meat was determined using near-infrared spectroscopy method. The measurement used a Food Scan Meat Analyzer [10]. Determination procedure: The samples (milled) were ground (milled) using a meat grinder; weighed (±30 g); and put into the cup/sample cups (diameter 15 cm) and then the surface was leveled (the surface was sealed); next, the connected computer with the foods scan tool was turned on; the icon for food scan was clicked or “the menu” was selected, and the food scan program was launched; and the configuration was selected by specifying parameters of the test including fat, protein, collagen, and water with the wavelengths set up between 800 and 1400 nm. The next stage was that the sample cup was inserted into the food scan space; the food scan analysis tool was selected and activated by pressing “Run/On.” After 15 min, the food scan analysis had been detected and had read the average moisture, protein, fat, and collagen in % units. In the end, the sample was given a unique code and file that had been read was saved. The measurement of the meat cholesterol levels was done by utilizing the Liebermann–Burchard Method [11]; and reading using a spectrophotometer at a wavelength of 680 nm.

### Determining the physical quality of meat

1. Determining water holding capacity (WHC) [12]. The stage of measurement began with determining free water content (FWC). Measurement procedures: Sample with weight of ±0.3 g was placed on a filter paper. Two plates were pressed under a load of 35 kg for ±5 min. The image area was established with plastic transparency and the specific area (cm^2^) was determined. The equation to calculate WHC was as follows: mgH_2_O=volume of the wet area (cm^2^)/0.0948. FWC (%) was calculated using the following equation: mgH_2_O/sample weight (mg)×100%. To determine the total water content (TWC), the following procedure was used: Filter paper (initial weight) and the sample weight±1 g (sample weight) were weighed and then wrapped with filter paper. Next, preheat the oven, and then heat the sample at 110°C for 8 h. The sample was weighed after being removed from the oven (final weight). The TWC (%) was calculated using the following equation: Sample weight−(final weight−initial weight)/sample weight (mg)×100%. The WHC (%) was calculated using the following equation: TWC (%)−FWC (%).
2. Determination of meat tenderness (kg/cm^2^) (Shear force Warner-Bratzler method) [12,13]. Measurement procedure: Sample of 20 g was weighed and placed on polypropylene plastic and packed with a vacuum pack. The sample was heated in a water bath at 80°C for 30 min. Next, the sample was removed from the water. After cooling it down, determined a sample field of the size of 1.5×0.67 cm or 1×1 cm or form a tube measuring a certain area in the direction of the fibers of the meat. The test sample plate was then placed on the tool with the fiber direction in a transverse position, and turn on the Warner-Bratzler shear machine. The tool cut the meat fibers. The measurement result was noted. Finally, the procedure was repeated 2 or 3 times.
3. Determination of the degree of acidity (pH) [12]. Measurement procedure: The samples of meat were mashed by chopping or using a meat grinder. As much as, 2 g of the mashed/ground meat was weighed for a sample and then diluted with 18 mL of distilled water, stirred/mixed until homogeneous, and finally filtered. The following steps were done using a pH meter calibrated using buffer solutions of pH 4 and pH 7. The filtrate samples were measured by a pH meter with specification HI 9811XPiccolo, Hanna Instrument, and the measurements were recorded. Using the samples, measurements were duplicated; after each pH measurement, the meter was checked for standardized calibration with solutions of buffer pH 7 and pH 4.
4. Determining the cooking loss [14]. Measurement procedure: As much as, ±20 g sample was weighed and placed in plastic polypropylene. The samples were packed using a vacuum pack, and then heated in a water bath at 80°C for 30 min. The cooking loss (%) was determined by the equation A−B/A×100%, where A and B, respectively, were the sample weights before and after the heating (g).

### Statistical analysis

Data were analyzed using variance according to the procedure of CRD. To obtain and fasten the analysis, the sofware IBM SPSS 19 Student Version was employed [15].

## Results

### Carcass and meat characteristics

The research result of cattle slaughter weight (Table-4) indicates that the provision of complete feed with different CP levels gave an insignificant difference in the slaughter weight. However, T2 treatment had the highest slaughter weight (239.000±21.280 kg), followed by T3 treatment (226.500±27.282 kg) and T1 treatment as the lowest (219.250±14.245 kg). Cattle carcass weight on T2 treatment was higher by 121.450±11.096 kg/cattle, followed by T3 (114.600±13.812 kg/head) and the lowest was T1 treatment (112.100±6.937 kg/head) (Table-4). In contrast, based on the carcass percentage, cattle on T1 treatment tended to be higher, followed by T2 and T3.

**Table-4:** Carcass characteristics of male Bali cattle fattened by complete feed which protein contains *Gliricidia sepium* leaves^1^

Variable	T_1_^2^	T_2_^2^	T_3_^2^
Initial weight (kg)^ns^	175.250±9.179	184.750±13.937	184.500±25.357
Final weight (kg)^ns^	229.500±11.690	253.750±13.326	240.750±31.889
BWG^3^ (kg)	54.250±4.717^a^	69.000±4.761^b^	56.250±11.870^a^
ADG^3^ (kg)	0.775±0.066^a^	0.985±0.071^b^	0.805±0.169^a^
FCR^3^	6.325±0.523^a,b^	5.835±0.369^a^	7.193±1.210^b^
Feed efficiency (%)	15.880±1.246b^a,b^	17.193±1.110^b^	14.178±2.201^a^
Slaughter weight (kg)^ns^	219.250±14.245	239.000±21.280	226.500±27.282
Hot carcass weight (kg)^ns^	112.100±6.937	121.450±11.096	114.600±13.812
Carcass percentage^ns^	51.140±0.512	50.820±1.385	50.61±1.595
Cool carcass weight^ns^	109.850±6.642	119.800±10.254	112.300±13.557
Meat weight (kg)^ns^	75.390±4.864	86.000±8.762	78.975±9.234
Meat percentage	68.615±0.620^a^	71.715±1.468^b^	70.350±0.570^b^
Bone weight (kg)^ns^	27.300±0.887	26.100±0.949	26.225±2.942
Bone percentage	24.893±1.094^b^	21.863±1.306^a^	23.375±0.556^ab^
Fat weight (kg)^ns^	6.170±0.386	6.928±0.565	6.253±0.813
Fat percentage^ns^	5.613±0.073	5.785±0.280	5.5725±0.380
The ratio of meat: bone	2.760±0.136^a^	3.293±0.266^b^	3.013±0.078^ba^
Non-carcass weight (kg)^ns^	107.150±7.450	117.550±11.138	111.900±14.224
Non-carcass percentage (%)^ns^	48.86±0.512	49.17±1.385	49.39±1.595

^1^Data is presented as average±SD, ^2^T_1_=Complete feed ration with CP 11%, TDN 72%, T_2_=Complete feed ration with 13% CP and TDN 72%, T_3_=Complete feed ration with 15% CP and TDN 72%, ^3^BW^0,75^=Metabolism body weight, ^3^BWG=Body weight gain, ^3^ADG=Average daily gain, ^3^FCR=Feed conversion ratio. ^a,b,c^Superscript which is different in the same line indicates difference (p<0.05), ns=Not significant

Non-carcass weight on T2 treatment cattle (117.550±11.138 kg/head) was higher than T3 treatment (111.900±14.224 kg/head), while the lowest was T1 treatment (107.150±7.450 kg/head). On the other hand, based on the on-carcass percentage, the cattle on T3 treatment had higher non-carcass percentage (49.39±1.595%) than T2 treatment (49.17±1.385%), while the lowest was T1 treatment (48.86±0.512%) (Table-4). The beef weighs and percentage, bone weight and fat weight of male Bali cattle given complete feed produced carcass weight (kg), which was relatively the same among T_1_, T_2,_ and T_3_ treatment. However, based on the percentage of bone produced, T1 cattle were higher (p<0.05) than T2 treatment, but relatively the same with T3 treatment. Likewise, the T2 treatment was relatively the same as T3 (Table-4).

The meat: A bone ratio of male Bali cattle fattening obtaining complete feed of T2 treatment with CP level of 13% and TDN level of 72% was higher (p<0.05) than T1 treatment which obtained complete feed with CP level of 11% and TDN level of 72%. However, T2 treatment was relatively the same as T3 treatment, which obtained complete feed with CP level of 15% and TDN level of 72%. Likewise, the T1 treatment was relatively the same as T3 treatment.

### Meat quality

#### Meat chemical quality

The research found that the use of complete feed, including protein, fat, water, collagen, and cholesterol in fattening male Bali cattle beef fattened, obtained relatively same chemical quality among the treatments given (Table-5). The beef protein content (%) of T1 was 23.22±0.85; T2 was 23.58±0.26; and T3 was 22.89±0.44. Meanwhile, the beef fat for each treatment of T1, T2, and T3 was 4.86±0.89; 4.77±0.65; and 5.61±0.47, respectively. The meat moisture content for T1 treatment was 73.32±0.64, T2 was 72.76±0.47, while T3 was 73.20±0.57. Furthermore, the collagen and cholesterol content of each treatment found to be 2.40±0.21 for T1; 28.79±4.42, 2.53±0.44; 29.77±3.16 for T2; and 2.45±0.20; 33.69±1.21 for T3 (Table-5).

**Table-5:** Meat chemical and physical quality of male Bali cattle fattened by complete feed containing protein from *Gliricidia sepium* leaves^1^.

Variable	T_1_^2^	T_2_^2^	T_3_^2^
Meat chemical quality (%)			
Protein^ns^	23.22±0.85	23.58±0.26	22.89±0.44
Fat^ns^	4.86±0.89	4.77±0.65	5.61±0.47
Water^ns^	73.32±0.64	72.76±0.47	73.20±0.57
Collagen^ns^	2.40±0.21	2.53±0.44	2.45±0.20
Cholesterol^ns^ (mg/100 g)	28.79±4.42	29.77±3.16	33.69±1.21
Meat physical quality			
Acidity^3 ns^	5.55±0.05	5.59±0.03	5.61±0.04
Tenderness (kg/cm^2^)^ns^	4.42±0.82	5.92±0.08	6.32±1.42
Cooking loss (%)^ns^	34.72±2.87	33.25±1.98	33.72±2.16
WHC (%)^ns^	29.54±5.69	32.34±26.26	29.76±1.42

^1^Data are presented as average±SD, ^2^T_1_=Complete feed Ration with CP 11%, TDN 72%, T_2_=Complete feed ration with 13% CP and TDN 72%, T_3_=Complete feed ration with 15% CP and TDN 72%, ns=Not significant

#### Meat physical quality

It was also found that the use of complete feed in fattening male Bali cattle obtaining relatively same physical quality for the treatment of acidity level, tenderness, cooking loss, and WHC (Table-5). Regarding the acidity level of beef for T1 was 5.55±0.05, T2 was 5.59±0.03, while T3 was 5.61±0.04. The beef tenderness (kg/cm^2^) for each treatment of T1, T2, and T3 was 4.42±0.82; 5.92±0.08; and 6.32±1.42, respectively. The cooking loss of the beef (%) for T1 treatment was 34.72±2.87; T2 treatment was 33.25±1.98; while for T3 treatment was 33.72±2.16. Finally, the WHC of the beef (%) for each treatment (T1, T2, and T3) was 29.54±5.69; 32.34±26.26; and 29.76±1.42, respectively.

## Discussion

### Carcass characteristics

#### Slaughter weight, carcass weight, and percentage

The application of complete feed with different CP level indicates insignificant differences in slaughter weight; however, T2 treatment tended to show a higher slaughter weight. This indicates that the effect given by the complete feed was the same in which the need for protein and energy in the form of TDN of Bali cattle aged 2-2.5 years has been fulfilled, which contributes to the synthesis of body tissues that are not much different to produce the same slaughter weight. A better level of feed intake in cattle will have a direct effect on increasing growth so that in a relatively short period of time, the beef growth is optimal and results in higher slaughtering weight [16].

The adequacy intake of protein and energy (TDN) in male Bali cattle fattened can improve the performance of cattle during the fattening. This was indicated by an increase in slaughter weight before slaughtered in the slaughtering house [4], and increasing carcass weight and dressing percentage in cattle [17].

Complete feed with high nutritional status will increase slaughter weight in a short time. In this study, the application of complete feed in fattening cattle was only for 90 days (3 months). This illustrates that complete feed is very efficient in shortening the fattening time so that it is economically profitable because of the savings in time and cost, especially the cost of the feed.

The high slaughter weight in T2 treatment resulted in higher carcass weight. In general, the results of this study indicate that the use of complete feed positively contributed to the synthesis of cattle body tissue, which, in turn, increased the carcass weight. The research feed formulation at the proportion of 11% CP and 72% TDN, 13% CP and 72% TDN, and 15% CP and 72% TDN (Tables-2 and 3), turned out to have a positive impact, thereby increased CP intake in T1, T2, and T3, respectively, by 14, 15, and 16%. On the contrary, cattle energy intake (TDN) in the three treatments was relatively low reaching 58%, 60%, and 54%, respectively. Nevertheless, the balance of CP and TDN in this study was good enough to produce high ADG as a result of high body tissue synthesis; which contributed to the production of high carcass weight as well. The balance of CP and energy (TDN) in cattle fattening is an important thing that needs concern. If the protein intake is high but TDN is low, it will affect the utilization of feed protein that is not optimal. The carcass weight of beef cattle is affected by muscle weight and muscle that determine the condition of the cattle body itself [18].

According to Choi *et al*. [19], carcass weight is strongly affected by slaughter weight, where the higher the slaughter weight, the more increase the carcass weight. The high carcass weight produced in this study was not always followed by a high percentage of carcasses because there were differences in the contents of the digestive tract. However, the carcass weight produced in this study was classified as good for Bali cattle with a slaughter weight of 219-239 kg. The cattle were considered good when producing carcasses with optimal quantity and quality.

The complete feed used in this study was composed of several feed ingredients such as field grass, cornmeal, *G. sepium*, rice bran, and bran pollard with adequate and sufficient palatable nutritional content. The quality of such feed can increase the intake and digestibility of feed in cattle that consume it to produce slaughter and carcass weight.

According to various reports, cattle carcass heat production is not the same in the use of different CP levels in different basic feeds. The use of CP at the level of 13% was optimal to produce higher carcasses in cattle. On the contrary, increasing CP concentration to 14.5% did not improve carcass performance. Cattle that obtained CP at 11.5% level also had lower carcass production [20]. The results of this study are slightly different from the opinion above related to the production of carcasses.

The results of this study also illustrated the different types of cattle, body weight, and ration feed composition. According to Bennett *et al*. [21], cattle that obtain forage feed in the final phase of fattening will have a lighter carcass weight compared to male cattle, which obtain concentrate feed in the same final fattening phase. When fattened cattle get high concentrations, it contributes to the production of higher rumen fermented propionic acid. As a result, the resulting blood glucose is higher to meet the needs of body tissue synthesis and increase carcass production [22].

The carcass weight produced from this study was higher than the report given by Suryanto *et al*. [23] in male Bali cattle given fermented cocoa pods weighing 98.28 kg. However, the weight and percentage of carcasses of male Bali cattle in this study were lower than the report given by Tahuk *et al*. [4] which gained rough weights ranging from 123.64 to 138.68 kg; and the percentage of carcasses ranged from 54.07% to 55.61%; and the report of Leslie *et al*. [24] which obtained a percentage of carcasses ranging from 57.17±2.43% to 59.02±1.08% in male Bali cattle fattening in oil palm plantations.

Likewise, the percentage of carcasses of Bali cattle in this study was lower when compared to the percentage carcasses of Simmental cattle that ranged 61.8±1.2-62.3±1.7% [17]. This shows that differences in breed and genetic herd determine the high or low of carcasses percentage produced by livestock. The high nutrient content and short time of this study provided an opportunity to increase the percentage of carcasses, according to Leo *et al*. [25]; Bali cattle have a potential carcass percentage of 53.94-59.02%.

#### Weight and non-carcass percentage

The results of this study indicate that the non-carcass weight produced in this study was still quite high. The Bali cattle were fasted for 12 h and it had not much impact on reducing the contents of the tract. Apart from slaughter weight and carcass weight, non-carcass weight is also closely related to the feed ingredients obtained and their digestibility. The low digestibility of feed contributes to the increased contents of the digestive tract so that the non-carcass produced is also higher. The digestibility of crude fiber of complete feed which was quite low is only around 22.420±6.031-24.665±5.793% (Table-4) contributing to the still high contents of the digestive tract of the cattle.

Besides, the use of complete feed impacted the accumulation of fat and the expansion of cattle body tissue so that the non-carcass weight component was even greater. Wet skin weight and head weight increase with the increase of status and slaughter weight due to the expansion of skin tissue and increasing body fat (BF), which is getting bigger [26]. The weight and non-carcass percentage in this study were higher than the report of Tahuk *et al*. [4], and the report of Suryanto *et al*. [27] in male Bali cattle fattening. This shows that differences in feed can contribute to different non-carcass production of livestock.

#### Component of carcass

The Bali cattle in T2 and T3 treatments had higher meat percentage because the slaughter weight and carcass weight produced were also higher than the T1 treatments. Furthermore, the results of this research illustrated that the use of complete feed ration formulation of 13% and 15% CP with 72% TDN (Tables-2 and 3) has been optimized to meet the needs of cattle both for basic living needs and production needs. The impact of meeting CP’s needs is an increase in cattle body tissue synthesis, especially muscle tissue. The increase in the proportion of beef was also affected by the weight and percentage of bone at T2 and T3, which was also lower at T1 treatment.

Meat produced by T2 treatment cattle reached 35.983% and T3 treatments reached 34.868% of slaughtering weight, higher than T1, which has 34.338% of meat from slaughtering weight. The proportion of beef in the three treatments was in the normal range because it still followed Soeparno opinion [28], stating that cattle can produce beef in the amount of 30-40% slaughter weight.

However, the meat beef weight, as well as the beef proportion from the slaughter weight in this research report, was lower than the report of Tahuk *et al*. [4] who had a meat weight ranging from 84.98 to 93.16 kg, with the percentage of meat from the slaughter weight ranging from 35.67% to 37.35% of male Bali cattle fattening on smallholder farms. Instead, the results of this research are relatively the same as the report by Leslie *et al*. [24] who obtained meat percentage of male Bali fattening in oil palm plantations amounting to 68.19±1.96%-72.05±1.98%.

The proportion of bone results from studies in the cattle of T1 treatments was higher than T2 treatment because the proportion of meat produced was lower. The proportion of bones as a component of the carcass is generally and relatively constant when cattle have reached maturity. However, the weight or percentage of bone can increase or decrease if the meat and fat as the components of the carcass change in proportion. When it is related to this research, it can be seen that the proportion between meat and fat produced by cattle was not much different in the three treatment groups; consequently, the proportion of bone produced was relatively the same.

The percentage of bone produced in this study is not much different from the research reported by Ngadiyono *et al*. [16] who obtained a bone percentage of 24.24% and 22.35%, respectively, for the T0 and T1 groups in Ongole crossbreed cattle aged 1.5-2 years which obtained forage and concentrate ratio of 20:80 with an energy content of 72%; and the report of Tahuk *et al*. [4] which obtained bone percentages ranging from 23.21±1.12 to 24.34±1.28 in male Bali cattle fattening in Indonesian smallholder farms with different CP levels. Nevertheless, the percentage of bones in this study is higher than the report by Leslie *et al*. [24] who obtained bone proportions ranging from 12.56±1.45% to 23.56±1.47% in male Bali cattle fattening in oil palm farm.

The fat of carcass in the T1-T3 cattle groups was relatively the same. This condition illustrated that the response of treatment animals to the use of complete feed in fattening was not much different; consequently, the synthesis of BF tissue was also not much different. The use of the same energy in ration formulation of 72% TDN was sufficient to meet the basic living needs of the cattle so that excess energy was used to deposit fat tissue. Carcasses produced by sheep or cattle and pigs or chickens that receive high-energy feed will have more fat than carcasses of animals that are fed low-energy feed [28]. This research report also explained that Bali cattle tend to deposit BF when given high-energy feed.

The weight and percentage fat of carcass produced in this study are lower than the report of Tahuk *et al*. [4] who obtained carcass weights of 8.73±0.67-14.52±1.65, as well as fat percentages ranging from 7.06±0.23 to 10.53±0.74 in male Bali cattle fattened in Indonesian smallholder farms; report Leslie *et al*. [24] who obtained carcass fat from male Bali cattle fattening in oil palm plantations with a carcass fat range of 7.79±0.48%-16.67±1.82%.

The ratio of meat:bone of male Bali cattle fattening obtaining complete feed T2 and T3 treatments with CP level 13% and 15% with TDN 72% was higher than T1 treatments. This condition illustrates that the nutrient formulation in complete feed used has been optimized to increase muscle tissue synthesis as indicates by an increase in body weight gain, carcass production, and the amount of meat produced. When the synthesis of body tissues is high, the proportion of muscle tissue will also be high. The obtained meat/bone ratio, which was relatively similar between T1-T3 and T2-T3 treatments, was affected by the proportion of meat, fat, and bone which was not much different between the cattle groups. According to Soeparno [26], the meat:bone ratio is important to be concerned with cattle fattening because it has to do with the degree of muscularity in the carcass and the amount of meat produced. Conversely, muscle in thick carbohydrates with low fat can increase the meat:bone ratio.

The ratio of meat:bone of male Bali cattle in this study was higher than the report of Tahuk *et al*. [4] who obtained a meat:bone ratio range of 2.69±0.22-2.90±0.14; but almost the same as the report of Suryanto *et al*. [27] with the meat:bone ratio obtained ranges 2.9:1-3.9:1. The difference in the ratio of meat and bones between several studies was affected by differences in physical form and quality of feed used, carcass production, meat and fat, and the proportion of bone from the cattle slaughtering.

### Beef quality

#### Chemical meat quality

Protein content

The meat protein content (%) of male Bali cattle obtaining complete feed from *G. sepium* leaves as the protein source was relatively the same among the treatments provided and was still at the normal range (Table-5). The use of complete feed at CP level of 11%, 13%, and 15% with TDN content of 72% (Tables-2 and 3) that produced relatively the same meat protein content level on male Bali cattle indicates that the complete feed was optimal to increase the meat protein. It is in line with the previous research conducted by Kučević *et al*. [17], who obtained indifferent results stating that the beef protein content was not affected by the gender of the cattle, feed, or procedure of the beef cattle fattening.

Total N level (% fat-free) of meat lean from longissimus dorsi muscle was theoretically 3.6%. When it is converted to CP, then it will reach 22.5% [29]. According to Soeparno [26], the cattle which are growing will have increased synthesis rate and protein degradation, in which the protein synthesis rate of the beef vein will be more than the protein degradation. On the other hand, when the cattle are approaching maturity, then the synthesis rate and degradation will decrease; and further step is synthesis rate and degradation will reach low and balance value.

The meat protein content of previous research varied. Tahuk *et al*. [4] reported the meat protein content of male Bali cattle fattened range 21.21-21.71%; report by Ngadiyono *et al*. [16] with a range 21.77-22.43%; and report by Kučević *et al*. [17] on Simmental male cattle beef with a protein content range of 21.07±0.72%-21.38±0.68%. Compared to some of the reports above, then the protein content of the present research was higher. The different beef protein content in between research reports occurred due to several factors, including genetic and feed used.

Fat content

The relatively same fat of meat deposition on cattle obtaining complete feed with CP level of 11-15% indicates that the three ratio types were not far different from each other particularly the energy content. The ISO energy (TDN 72%) provided to three types of ratio in this research caused the fat synthesis to be relatively the same. This condition also illustrated that the energy need on the three cattle groups has been fulfilled so that the surplus was deposited into fat. Furthermore, the age of the three cattle groups has entered the maturity phase, thus the fat formation also increased. The beef fat content on Bali cattle is quite low and normal. According to Troy *et al*. [30], in general, lipid fraction in beef varies from 4% to 15% on fresh basis depending on several factors, including genotype, feeding regime, and meat cut.

The various fat of meat level can be affected by the family, age, species, muscle location, and feed [29]. The energy concentration of the feed and/or protein/energy ratio in feed also the use of volatile fatty acids especially propionate for gluconeogenesis which can cause meat chemical component changes especially on the carcass fat content [26].

According to Park *et al*. [31], the genetic factors which affect the intramuscular fat (IMF) deposition on cattle are the cattle family, gender, and heritability factor, while the management factors which affect the IMF are the cattle age, castration, age, and weight during the cutting as well as the other nutritional and environmental factors. Big type cattle cut on age exceeding the adult age will result in constant or decreased protein growth level. It is then dominated by fat deposition [26].

The fat of meat content of male Bali cattle fattened fed 100% forage and digestible carbohydrate supplementation was 3.03±0.76%-4.68±1.51% [4]. Otherwise, the fat content of male Ongole cattle crossbreed which obtained two different concentrates treatments was at the range of 0.83-1.64% [16]. Thus, the fat of meat content in this research is higher than the fat content in the previous research explained. The present research result proved that the provision of complete feed on male Bali cattle fattened positively contributed to the increase of its fat of meat. Furthermore, the high content of beef fat indicates that Bali cattle have a tendency to deposit the fat if it obtains high-quality feed.

Water content

The biggest chemical component of fresh meat is water. The water content affects the quality of beef especially juiciness, tenderness, color, and taste [26]. General research found that the water content of beef among the treatments given is relatively the same (Table-5). Such condition proved that the use of complete feed given to male Bali cattle fattened contributes positively on the meat water content. The water content of the beef which is relatively the same is in line with the beef fat content which is also not far different among the treatments provided. Theoretically, there is a negative relationship between the meat water content and the meat fat content. If the fat content of meat increases, the water content of meat usually decreases [26].

The three cattle groups in this research have a higher water content that the result of research conducted by Tahuk *et al*. [4], who found that the beef water content of male Bali cattle fattened using forage and supplemented by digestible carbohydrate had water content of 71.73±0.52-72.75±0.54^b^ or 72.15±0.53 at the average; but relatively the same as the report by Kučević *et al*. [17] who obtained water content range of 72.11±1.43%-74.54±1.32% on Simmental male and female cattle fattened. The water content of this previous research also almost the same as research result reported by Ngadiyono *et al*. [16], who found that the water content of *L. dorsi* and BF muscle on male Ongole crossbreed cattle obtaining different concentrate (T_0_ and T_1_) was 74.26% and 73.48%, and 73.48% and 72.96%. According to Lawrie [29], the water content of mammalian tendons after rigor mortis can reach 75%. Therefore, the water content of the beef in this research was at the normal range. The factors affecting the different water content of meat cattle are genetic, feed used, variety in raising procedure, and the cattle gender [17], and variety of fat deposit in cattle beef fattened [29].

Collagen content

The distribution of meat collagen of the three male Bali cattle groups in this research was relatively low and same among the treatment. The low meat collagen indicates that the use of complete feed to fatten the male Bali cattle positively contributed to the beef quality. The factors affecting the beef collagen are the fat content, where the beef fat content dissolves or decreases the collagen content. In this research, beef collagen can be decreased due to the beef fat content which was quite high. Furthermore, the factors affecting the meat collagen level are the gender and muscle on the same carcass [29], age [29,32], cattle family, feed, and pattern of cattle raising [32,33].

The relatively same collagen content was also due to the number of cross ties owned by the cattle beef, which was not far different, in which the amount of cross ties also affected by the cattle activities. In this research, the cattle were caged so that the activities were limited; thus, the collagen formation of the three cattle groups was not significant. The quite low collagen caused the beef produced by the cattle fattened for having good tenderness (Table-5). The beef collagen in this research was higher than the research result reported by Tahuk *et al*. [4], who found that the male Bali cattle raised in local farms have collagen of 1.56±0.14-1.72±0.09.

### Cholesterol

Cholesterol content in beef has become a serious concern of consumers because of the impact on health problems caused. Therefore, the presence of a high level of lipids in beef has been a topic of discussion for beef consumers because of their associated health implications [30].

In this research, the male Bali cattle produced beef cholesterol, which was not far different among the treatments, in which they were still at the range of 28.79±4.42-33.69±1.21 mg/100 g (Table-5). The cholesterol content of the male Bali cattle fattened using complete feed was lower than the previous research reported. According to Suryanto *et al*. [27], Bali cattle, which were given fermented cacao produced cholesterol at the range of 38.75±2.63-42.00±4.97 mg/100 g. Furthermore, other researcher reported that the beef cholesterol produced was 36-53 mg/100 mg on *longissimus* muscle and 50-55 mg/100 g on femoris biceps muscle [34]. According to Morales *et al*. [35], cholesterol of beef cattle can be increased based on the raising pattern and feed given. The cattle raised in the pasture was lower in cholesterol than the cattle raised through feedlot way.

This research found that the complete feed provided to the Bali cattle fattened in the form of protein source from *G. sepium* leaves positively contributed to decreasing beef cholesterol. Furthermore, the cattle used in this research were still young; thus, it was suspected to affect the formation of beef cholesterol. Such condition was seen from the fat deposition, which was not far different from each other group in this research. The age was found to significantly affect beef cholesterol. The older the cattle, the higher the cholesterol compared to the younger cattle [36].

Summarizes from various references, the differences in cholesterol content among species were generally explained by variations in absorption and biosynthesis of cholesterol, lipoprotein metabolism, diet, muscle fiber type distribution, genetic variation, subcutaneous and IMF, and body weight [34].

### Physical meat quality

#### Acidity degree (pH)

The male Bali cattle involved in this research relatively produced the same acidity degree (pH) among the treatments and were still at normal range. This illustrates that the provision of complete feed with different levels of CP on male Bali cattle fattened positively contributed to the pH of the cattle. It was suspected that the muscle glycogen reserves produced by the cattle from the groups were not different from each other causing the beef pH to be relatively the same. The general ultimate pH was at the range of 5.5-5.8 and was affected by the muscle glycogen reserves during the cutting. The more the glycogen reserves during the cutting, the lower the ultimate pH achieved [28].

This research found that the acidity degree of this research is not far different from previous research result reported by Tahuk *et al*. [4], who obtained meat pH at the range of 5.65±0.21-5.74±0.14 on male Bali cattle fattened in Indonesian smallholder farms; the research reported by Martiana *et al*. [37] who obtained meat pH of Bali cattle at the range of 5.43±0.13-5.57±0.07; and the result of the research reported by Pogorzelska-Przybyłek *et al*. [38] who obtained meat pH at the range of 5.52-5.57 on several beef cattle family with various age.

Even so, the pH value in this research is lower than the research result reported by Leslie *et al*. [24], on male Bali cattle in Malaysia which obtained natural forage and legumes in oil palm plantation producing pH of 6.33±0.08-6.85±0.04, and those who were raised using feedlot at the range of 6.02±0.11-6.79±0.15. The final acidity degree of beef is a clue to know the quality of beef. The beef that has normal pH at the range of 5.5-5.7 is red [29].

#### Cooking loss

The observation conducted in this research found that the treatment by giving complete feed with different levels of CP on male Bali cattle was relatively the same and quite low. This condition illustrates that male Bali cattle fattening using complete feed can increase the quality of the meat. This indicates that the nutrition of the beef was still protected during the cooking process. The low cooking loss in this research is related to the WHC, which was quite high and the ultimate pH of beef, which was optimal from the three cattle groups; those were 29.54±5.69%-32.34±26.26% and 5.55±0.05-5.61±0.04.

The use of complete feed with CP level of 11-15% was generally seen to be optimal in producing qualified beef to meet the consumers’ need. Complete feed with such formulation was sufficient to fulfill the energy need of the cattle and increase the IMF synthesis (marbling) on beef. The role of IMF is to protect the liquid produced during the cooking process so that the beef nutrition can be kept well.

Production of liquid during the cooking process can be inhibited by the formation of high IMF. Thus, the value of cooking loss of beef is significantly related to the type of feed given [28]. Forage with digestible energy given to cattle will cause low marbling so that the cooking loss will increase. There was no significant effect of family differences and age of the cattle on the cooking loss of the beef of male Bali cattle fattened using the complete feed, according to Biraima *et al*. [39].

Factors affecting the cooking loss are the high number of cellular membrane damage, the high production of water produced by the meat, the storage age of the meat and the ability of the meat to hold water, and the protein degradation [40]; as well as the length of sarcomere muscle fibers, the length of muscle fiber cut, the myofibril contraction status, and size and weight of the beef as well as the meat cross-section. In addition, there is a relationship between the pH value of the beef and the cooking loss [28]. Meanwhile, Pogorzelska-Przybyłek *et al*. [38] reported that different family and age of the cattle did not affect the cooking loss of beef cattle.

In this research, the cooking loss was lower than meat cooking loss of male Bali cattle in Malaysia, which was at the range of 33.25±0.97%-38.06±1.59% which obtained natural forage and legume in palm oil plantation using integrated raising system; and feedlot system at the range of 35.38±1.71%-42.48±1.21% [24]; as well as the research result reported by Tahuk *et al*. [4] who found that the cooking loss of Bali cattle raised in Indonesian smallholder farms which was at 37.36±2.31%-40.50±1.11%. On the other hand, the cooking loss of this research is almost the same as the result reported by Pogorzelska-Przybyłek *et al*. [38], who obtained cooking loss at the range of 32.89-33.90% at several beef cattle.

#### Tenderness

Male Bali cattle fattened using complete feed produced quite tender meat at the range of 4.42±0.82-6.32±1.42 (kg/cm^2^) for all treatment groups (Table-5). This indicates that the use of complete feed positively contributed in improving the quality of meat Bali cattle. According to Scaglia *et al*. [41], the fattening of beef cattle using forage or concentrate will produce carcass with tenderness not far different from each other.

Based on several references, the shear force value range on the meat tenderness is divided into three categories, which are 0-3 for tender, 3-6 for quite tender, and 6-11 kg/cm^2^ for rough. The shear force measurement producing value of more than 11 indicates that the meat is very rough to be eaten by human [42]. The higher the breaking power means the higher the roughness of the meat, on the other hand, the lower the breaking power means the more tender the meat [28]. Therefore, the beef of cattle group of T_1_ to T_3_ is included to quite tender beef.

The tender beef produced in this research has a relationship with other variables such as the higher WHC, the lower cooking loss from the three cattle groups. Theoretically, the tenderness of meat is affected by three beef components; those are myofibrils structure and its contraction status, connective tissue content and its cross ties level, WHC, and the juiciness of the meat [28]. Meanwhile, the age and the type of muscle did not affect the beef cattle tenderness significantly [32,43]; other factors such as the cattle family and feeding time also did not significantly affect the tenderness of the beef as reported by Nian *et al*. [32].

The quite tender meat of Bali cattle on the three treatment groups was also caused by the IMF content, which was quite high. Observation conducted on *Bos taurus* and *Bos indicus* cattle indicates that the increase of IMF also increases the tenderness of the beef [44]. In addition, the collagen and fat content of the beef are relatively the same, so that it causes the tenderness to be quite the same as well.

Various research results reported that the collagen denaturation factor also determines the difference in meat breaking power. In addition, the natural characteristics of collagen fiber also affect its solubility [28]. The collagen cross ties also determine the meat tenderness where the meat collagen which has stabile hot cross-tied when heated will increase the density and roughness of the meat. On the other hand, the meat collagen which has unstable hot-ties, when heated it will increase the solubility level of the collagen so that the meat becomes tender [29]. Therefore, the quite tender of meat male Bali cattle obtained in this research was suspected to be caused by relatively the same cross ties and solubility level which was quite high on beef.

The beef tenderness obtained in this research is higher than the research reported by Tahuk *et al*. [4], who was 5.58±0.79-8.80±0.86 kg/cm^2^ on male Bali cattle raised in Indonesian smallholder farms; the meat tenderness of Bali cattle raised in Malaysia which was 6.09±0.70-10.25±1.64 kg/cm^2^ obtained natural forage and legume in palm oil plantation in integrated raising system, as well as the feedlot raising system at the range of 6.76±0.78-12.55±1.42 kg/cm^2^ [24].

#### The WHC

WHC variable significantly determines the meat quality. The decrease of WHC causes the loss of protein and vitamin along with the water so that it produces low-quality beef processed product [45]. The meat of male Bali cattle fattened using complete feed on the three treatment groups produced WHC which was quite high and at normal range (Table-5). This indicates that the ratio given to the three cattle groups was adequate in terms of the quality and quantity so that it positively contributed to the WHC of the beef.

The previous research reported that pH has an important role in determining the normality of WHC. The decrease of carcass pH (postmortem) will affect the WHC [29]. In this research, the beef pH obtained was at normal range, thus WHC obtained was also at normal range.

In addition to pH, the age of cattle, which was quite young also suspected to affect the relatively same WHC among the treatment groups. According to Biraima *et al*. [39], the difference of age can affect the WHC in which the cattle, which is at old age will have lower WHC. Thus, according to the opinion above, WHC produced in this research was quite high on all treatment groups because the cattle involved were still relatively young.

## Conclusion

The application of complete feed with protein sources from *G*. *sepium* with CP and TDN content of 13 and 72% can increase the carcass characteristics of male Bali cattle fattened seen from the percentage of carcasses than the use of CP 11% and 15% with TDN 72%. Nevertheless, other variables, including meat quality do not show significant differences between the three treatments.

The using complete feed with 15% CP and 72% TDN is no longer effective in male Bali cattle fattened because it shows carcass percentage that is no better than the use of CP and TDN at 13% and 72% levels. It is proven that the use of complete feed with the right protein and energy formulations contributes positively to the male Bali cattle fattened.

## Authors’ Contributions

PKT contributed to experimental design of the research, data analysis, reporting, and drafting of the article. ORN contributed to supervise the research and data analysis. GFB conducted the processing of complete feed and applications, and data collection. All authors have read and approved the final manuscript.
